# Soil Microbial Responses to Elevated CO_2_ and O_3_ in a Nitrogen-Aggrading Agroecosystem

**DOI:** 10.1371/journal.pone.0021377

**Published:** 2011-06-22

**Authors:** Lei Cheng, Fitzgerald L. Booker, Kent O. Burkey, Cong Tu, H. David Shew, Thomas W. Rufty, Edwin L. Fiscus, Jared L. Deforest, Shuijin Hu

**Affiliations:** 1 Department of Plant Pathology, North Carolina State University, Raleigh, North Carolina, United States of America; 2 Plant Science Research Unit, United States Department of Agriculture, Agricultural Research Service, Raleigh, North Carolina, United States of America; 3 Department of Crop Science, North Carolina State University, Raleigh, North Carolina, United States of America; 4 Department of Environmental and Plant Biology, Ohio University, Athens, Ohio, United States of America; J. Craig Venter Institute, United States of America

## Abstract

Climate change factors such as elevated atmospheric carbon dioxide (CO_2_) and ozone (O_3_) can exert significant impacts on soil microbes and the ecosystem level processes they mediate. However, the underlying mechanisms by which soil microbes respond to these environmental changes remain poorly understood. The prevailing hypothesis, which states that CO_2_- or O_3_-induced changes in carbon (C) availability dominate microbial responses, is primarily based on results from nitrogen (N)-limiting forests and grasslands. It remains largely unexplored how soil microbes respond to elevated CO_2_ and O_3_ in N-rich or N-aggrading systems, which severely hinders our ability to predict the long-term soil C dynamics in agroecosystems. Using a long-term field study conducted in a no-till wheat-soybean rotation system with open-top chambers, we showed that elevated CO_2_ but not O_3_ had a potent influence on soil microbes. Elevated CO_2_ (1.5×ambient) significantly increased, while O_3_ (1.4×ambient) reduced, aboveground (and presumably belowground) plant residue C and N inputs to soil. However, only elevated CO_2_ significantly affected soil microbial biomass, activities (namely heterotrophic respiration) and community composition. The enhancement of microbial biomass and activities by elevated CO_2_ largely occurred in the third and fourth years of the experiment and coincided with increased soil N availability, likely due to CO_2_-stimulation of symbiotic N_2_ fixation in soybean. Fungal biomass and the fungi∶bacteria ratio decreased under both ambient and elevated CO_2_ by the third year and also coincided with increased soil N availability; but they were significantly higher under elevated than ambient CO_2_. These results suggest that more attention should be directed towards assessing the impact of N availability on microbial activities and decomposition in projections of soil organic C balance in N-rich systems under future CO_2_ scenarios.

## Introduction

Soil microbes critically affect plant and ecosystem responses to climate change by modulating organic C decomposition and nutrient availability for plants. Experimental evidence accumulated over the last several decades has clearly shown that climate change factors such as CO_2_ enrichment in the atmosphere can significantly alter plant growth [1], [2] and the availability of organic C, N and cation nutrients for microbes [3], [4], [5]. Ozone is a greenhouse gas with demonstrated inhibitory effects on plant growth and resource allocation belowground [6], [7]. Although less well-studied, O_3_ is considered to have an impact on soil microbial processes [6]. Alterations in soil microbes can, in turn, profoundly influence soil C processes and the long-term potential of terrestrial ecosystems as a C sink to mitigate anthropogenic sources of atmospheric CO_2_. However, predicting what these changes will be is hampered by our limited understanding of the underlying mechanisms by which soil microbes respond to altered resource availability.

The current prevailing hypothesis, building on the assumption that soil microbes are generally C limited [8], predicts that elevated CO_2_ increases soil microbial biomass and activities due to enhanced soil C availability [9], [10], [11], whereas O_3_ reduces them due to lower C allocation belowground [6], [12], [13]. This broad hypothesis has been extensively tested over the past two decades for CO_2_ but less so with O_3_ [4], [10], [12], [13], [14], [15], [16]. Though C availability to microbes has been commonly reported to increase under elevated CO_2_ [5], [17], [18] and to decrease under elevated O_3_ [6], [13], [19], results of soil microbial responses to elevated CO_2_ and O_3_ have been inconsistent [6], [11], [20], [21]. In a meta-analysis study, de Graaff *et al*. (2006) found that elevated CO_2_ increased microbial biomass C and microbial respiration by 7.7% and 17.1%, respectively, across 40 studies that mainly included herbaceous species. In the meantime, Hu *et al*. (2006) reviewed 135 studies examining elevated CO_2_ effects on a suite of soil microbial parameters such as microbial biomass and respiration and found that microbial biomass C and microbial respiration increased under elevated CO_2_ in 19 of 40 studies and 20 of 38 studies, respectively, but remained unchanged or even decreased in the remainder. Despite considerable efforts in the past two decades, there is a lack of conceptual understanding of why and how these inconsistencies in CO_2_ and O_3_ effects on microbes occur.

Soil microbial responses to elevated CO_2_ can also be influenced by CO_2_-induced alterations in soil moisture [9], [22], grazing activity of soil animals [23], and soil nutrient availability [4], [9]. Such mechanisms could operate either singly or in combination with changes in soil C availability. In particular, CO_2_-induced alteration in the stoichiometery of available C and N has been proposed to be a primary control over microbial responses to elevated CO_2_ [4], [9], [24]. Soil N availability may influence microbial responses to elevated CO_2_ by affecting both physiological activities and the community structure composition of microbes [9], [25]. When soil N was limiting, competitive plant N uptake can significantly reduce soil N availability for microbes under elevated CO_2_, limiting microbial decomposition over the short-term [4]. Conversely, high N availability in soil often increases microbial activities [26], [27] and favors bacteria over fungi [28], [29]. Yet most studies that examined microbial responses to elevated CO_2_ were conducted in N-limiting forest and grassland ecosystems [20], [21]. It remains unclear how soil microbes respond to elevated CO_2_ and O_3_ in N-rich or N-aggrading agroecosystems.

Many crop plants, particularly C_3_ crops, are usually responsive to elevated CO_2_ and O_3_ [2], [30]. For instance, it has been estimated that elevated CO_2_ alone increased the shoot biomass of soybean and wheat by 48% and 16%, respectively [2], [31], but elevated O_3_ reduced them by 21% and 18% [32], [33]. Also, elevated CO_2_ has been shown to ameliorate O_3_ effects on plants by reducing O_3_ uptake and increasing C assimilation rates [30], [32], [34]. However, whether the CO_2_- and O_3_-induced changes in plant biomass translate into alterations in soil C sequestration depends largely on the responses of soil microbial processes. Additionally, elevated CO_2_ significantly increased symbiotic N_2_ fixation in legumes such as soybean and peanut [35], [36], whereas elevated O_3_ tended to reduce it [36]. It has been suggested that high N availability in agro- and grassland ecosystems can sustain plant responses to rising CO_2_ over a long time frame and provide an opportunity for soil C sequestration in soil in a higher CO_2_ world [2], [37], [38], [39]. Convincing evidence is still lacking, but soil microbial responses may be indicative for understanding the long-term soil C dynamics in high N or N-aggrading ecosystems [27], [40].

In a long-term study examining climate change effects on soil C dynamics in a wheat-soybean agroecosystem with no-till practice, we continually monitored a suite of soil microbial parameters in response to elevated CO_2_ and O_3_ for more than four years. Because soybean and its symbiotic N_2_ fixation are sensitive to elevated CO_2_ and O_3_ [35], [36], we expected that elevated CO_2_ would enhance both C and N inputs belowground through increasing residue returns, while elevated O_3_ would offset this CO_2_ effect. Also, we expected that the stoichiometry of available C and N for microbes might change over time as a portion of residue C was mineralized and released back to the atmosphere as CO_2_ while a large proportion of residue N was retained in the system. Consequently, alterations in C and N availability for microbes induced by elevated CO_2_ and O_3_ may further affect microbial biomass and activities over time, and possibly induce a shift in the microbial community structure. Therefore, our specific objectives were to: 1) document the time-course of CO_2_ and O_3_ effects on microbial biomass, activities and community structure; and, 2) examine how changes in microbial parameters were related to residue inputs and soil C and N availability.

## Materials and Methods

### Site description

We initiated a long-term field experiment in May 2005 to investigate the response of a wheat-soybean rotation agroecosystem to elevated atmospheric CO_2_ and O_3_ using open-top field chambers (OTC). The experimental site is located at the Lake Wheeler Experimental Station, 5 km south of North Carolina State University, Raleigh, North Carolina, USA (35°43′N, 78°40′W; elevation 120 m). Annual mean temperature is 15.2°C and annual mean precipitation is 1050 mm. The field had been left fallow for eight years prior to this study. Before CO_2_ and O_3_ treatments were initiated, the soil was repeatedly turned-over using a disc implement and rotovator. The soil is an Appling sandy loam (fine, kaolinitic, thermic Typic Kanhapludult), well drained with a pH of 5.5, and contained 9.0 g C and 0.86 g N kg^−1^ soil when the experiment started.

This experiment was a 2×2 factorial design with four treatments randomly assigned into four blocks. Four different trace-gas treatments were: (*a*) charcoal-filtered air and ambient CO_2_ (CF); (*b*) charcoal-filtered air plus ambient CO_2_ and 1.4 times ambient O_3_ (+O_3_); (*c*) charcoal-filtered air plus 180 µl l^−1^ CO_2_ (+CO_2_); and (*d*) charcoal-filtered air plus 180 µl l^−1^ CO_2_ and 1.4 times ambient O_3_ (+CO_2_+O_3_). The seasonal daily average concentrations of CO_2_ and O_3_ over the experimental duration are shown in Table 1. The purpose of filtration of ambient air with activated charcoal was to reduce the concentrations of ambient O_3_ to levels considered nonphytotoxic to soybean and wheat plants. Ozone was deemed as a major air pollutant in this area, while other air pollutants such as NO_2_ and SO_2_ were below the phytotoxic levels at the experimental location [41].

**Table 1:** The seasonal daily average (12 h) CO_2_ and O_3_ concentrations at canopy height during the 4-year period.

Crop	CO_2_ (µl l^−1^)		O_3_ (nl l^−1^)	
	Ambient	Elevated	CF	Elevated
Soybean	376.0±0.4	555.0±0.7	19.9±0.3	65.7±0.4
Wheat	388.0±0.4	547.0±0.5	20.7±0.2	49.8±0.3

CF: charcoal filtered air. Values are mean ± s.e.m.

Soybean [cv. CL54 RR (Year 1), Asgrow 5605 RR (Years 2 and 3) and SS RT5160N RR (Year 4)] was planted each spring followed by soft red winter wheat (Coker 9486) in the fall using no-till practices. Plants were exposed to reciprocal combinations of CO_2_ and O_3_ within cylindrical OTCs (3.0 m diameter×2.4 m tall) from emergence to physiological maturity. Carbon dioxide was released from a 14-ton liquid-receiving tank 24 h daily and monitored at canopy height using an infrared CO_2_ analyzer (model 6252, Li-Cor Inc. Lincoln, NE, USA). Ozone was generated by electrostatic discharge in dry O_2_ (model GTC-1A, Ozonia North America, Elmwood Park, NJ, USA) and dispensed 12 h daily (08:00–20:00 hours Eastern Standard Time) in proportion to concentrations of ambient O_3_. The O_3_ concentration in the chambers was monitored at canopy height with a UV photometric O_3_ analyzer (model 49, Thermo Environmental Instruments Co., Franklin, MA, USA). During wheat growing seasons, each plot initially received 48 g NH_4_NO_3_ (equivalent to 24 kg N ha^−1^) in November each year, followed by an additional input of 192 g NH_4_NO_3_ (equivalent to 96 kg N ha^−1^) in March. Plots were treated with lime, K and P in November during the experiment according to soil test recommendations. During soybean growing seasons, plants were irrigated with drip lines to prevent visible signs of water stress, but no additional N fertilizers were applied. Upon senescence of the plants, all aboveground plant biomass in each chamber was harvested. Soybean plants were divided into leaves, stems, husks and seeds, while wheat plants were separated as straw, chaff and seeds, then dried and quantified. Afterward, residues other than seeds were uniformly returned to their corresponding treatment plots and evenly distributed on the soil surface.

### Soil sampling

The chamber plot was divided into two parts: the sampling area (an inner circular area with a diameter of 2.4 m) and the border area (for purpose of reducing chamber effects, 0.3 m in width). To avoid taking soil samples from the same location, the sampling area was divided into 448 small subplots (10×10 cm). Soil sampling locations were determined using a random number generator and each subplot was sampled only once. In June and November of each year (Year 1–Year 4), corresponding to harvest time for each crop, we used a 5-cm diameter soil corer to take three soil cores to 20 cm depth in the center of three pre-determined subplots from each chamber. Three additional soil cores were immediately taken from the border areas to fill holes in the sampling areas. Sample holes in border areas were refilled by soil cores taken just outside of each chamber. Soil cores were separated into 0–5 cm, 5–10 cm and 10–20 cm depth fractions. Core sections were then pooled by the depth fraction into three soil samples per chamber. Soil samples were also collected at the mid-growing season to check whether microbial parameters were significantly different from those obtained at the end of the growing season. Soil samples (0–5 and 5–10 cm) were collected in each April of the first two years and each August of the last two years, corresponding to the maximal physiological activity of wheat and soybean plants, respectively. All samples were sealed in plastic bags, stored in a cooler and transported to the laboratory.

Field moist soils were mixed thoroughly and sieved through a 4-mm mesh within 24 hours of the field sampling and all visible residues and plant roots were carefully removed. Subsamples (∼20 g) were then taken immediately; frozen and stored at −20°C for the phospholipid fatty acid (PLFA) analysis and the rest of soils were stored at 4°C for other microbial and chemical analyses. A 10-g subsample was oven-dried at 105°C for 48 h and weighed for the determination of the water content. All the soil and microbial data were calculated on the dry weight basis of soils.

### Sample analyses

#### C and N contents in plant residues and soils

Air-dried subsamples of aboveground plant components (stems, leaves, husks and seeds of soybean; straw, chaff and seeds of wheat) were ground in a Tecator Cyclotec mill fitted with a 1-mm screen (FOSS, Eden Prairie, MN, USA). Soil samples were ground into fine powder using an 8000-D Mixer Mill (SPEX CertiPrep Inc. Metuchen, NJ, USA). The C and N concentrations in various plant components and in soil were determined with a CHN elemental analyzer (Carla Erba and model 2400, Perkin Elmer Co., Norwalk, CT, USA). Aboveground residue C and N inputs to soil were calculated by adding up the C and N, respectively, in all aboveground plant components except for seeds.

#### Symbiotic N_2_ fixation in soybean

Soybean N_2_ fixation was estimated using the conventional N accumulation method [42]. To estimate total N_2_ fixation by soybean in each season, we first estimated total biomass N in wheat and soybean, respectively. Total aboveground plant N was calculated by directly adding up the N in all plant components. Root biomass was estimated by using the fixed root∶shoot ratios of wheat (0.07) and soybean (0.22) according to the literature [2], [31], [43], [44], [45]. We also assumed that the C∶N ratio of roots was the same as that of shoots [46]. Then, we used wheat in the following season as the nonfixing plant to estimate total N fixed by soybean in each season by subtracting total N in wheat plants from total N in soybean plants on a per chamber basis. Further, the CO_2_ effect on N_2_ fixation was estimated by subtracting total N in soybean in ambient CO_2_ from elevated CO_2_. Although wheat has been often used as a non-fixing control plant [42], we realized that this method does not provide an exact estimate of N_2_ fixation by soybean plants in the field. Using wheat plants as the non-fixing control in our system should provide a conservative underestimate of soybean N_2_ fixation because: 1) N inputs to soil through soybean root exudates and fine root turnover were not considered; 2) inorganic N fertilizers (120 kg N ha^−1^) were applied for wheat; and 3) wheat should have also obtained significantly higher amounts of N from the mineralization of soybean residues. In addition to estimating N_2_ fixation, changes in total soil N over time were documented by comparing the soil N content (0–5 cm soil layer) at the end of the fourth year to the pretreatment soil N content (0–5 cm soil layer).

#### Soil microbial biomass C and N

Soil microbial biomass C (MBC) and biomass N (MBN) were determined using the fumigation-extraction method [47]. Twenty-g dry weight equivalent soil samples were fumigated with ethanol-free chloroform for 48 h and then extracted with 50 mL of 0.5 M K_2_SO_4_ by shaking for 30 min. Another 20-g sample of non-fumigated soil was also extracted with 50 mL of 0.5 M K_2_SO_4_. Soil extractable organic C in both fumigated and non-fumigated K_2_SO_4_ extracts was measured using a TOC analyzer (Shimadzu TOC-5050A, Shimadzu Co., Kyoto, Japan). Soluble inorganic N in the extracts of fumigated and non-fumigated soils was quantified on the Lachat flow injection analyzer (Lachat Instruments, Milwaukee, WI, USA) after digestion with alkaline persulfate [48]. The differences in extractable organic C and inorganic N between fumigated and non-fumigated soils were assumed to be from lysed soil microbes. The released C and N were used to calculate MBC and MBN using a conversion factor of 0.45 (*k*_EC_) and 0.45 (*k_EN_*), respectively [47], [49].

#### Soil extractable C and N

The concentration of organic C in non-fumigated soil extracts was used to represent soil extractable C. The extractable inorganic N referred to the sum of NH_4_^+^-N and NO_3_^−^-N in non-fumigated soil extracts.

#### Soil microbial respiration

We determined soil heterotrophic respiration using an incubation-alkaline absorption method [50]. In brief, 20-g dry mass equivalent soil samples were adjusted to moisture levels of around 60% water holding capacity, placed in 1-L Mason jars, and then incubated at 25°C in the dark for 2 weeks. Respired CO_2_ was trapped in 5 mL of 0.25 M NaOH contained in a beaker suspended in the jar. After the first week incubation, NaOH solutions were replaced with fresh solutions. The CO_2_ captured in the NaOH solution was titrated with 0.125 M HCl to determine the amount of CO_2_ evolved from the soil. Soil microbial respiration (SMR) rate was expressed as mg CO_2_ kg^−1^ soil d^−1^ by averaging the data across two 1-wk incubations.

#### Net soil N mineralization

Potential N mineralization was determined following a 4-wk incubation at 25°C in the dark. Soil NH_4_^+^ and NO_3_^−^ in un-incubated and incubated subsamples (20-g each) were extracted with 50 mL of 0.5 M K_2_SO_4_ by shaking for 30 min. The concentrations of inorganic N were then measured on the Lachat flow injection analyzer. Net mineralized N (NMN) was determined by the difference in extractable total inorganic N (NH_4_^+^-N + NO_3_^−^-N) between incubated and un-incubated soil samples.

#### Phospholipid fatty acids

PLFAs were extracted following a procedure described by Bossio *et al*. (1998). Briefly, 10 g of freeze-dried soils (0–5 cm soil layer) were extracted using a solution containing CH_3_OH∶CH_3_Cl∶PO_4_^3−^ (vol/vol/vol 2∶1∶0.8). Solid phase extraction columns (Thermo Scientific, Vernon Hills, IL, USA) were used to separate phospholipids from neutral and glycol-lipids. The phospholipids were then subjected to an alkaline methanolysis to form fatty acid methyl esters (FAMEs). The resulting FAMEs were separated and measured using gas chromatography on a HP GC-FID (HP6890 series, Agilent Technologies, Inc. Santa Clara, CA, USA); peaks were identified using the Sherlock Microbial Identification System (v. 6.1, MIDI, Inc., Newark, DE, USA). We chose the following fatty acids, i14∶0, i15∶0, a15∶0, 15∶0, i16∶0, 16∶1ω7c, i17∶0, a17∶0, 17∶0cy, 17∶0, 18∶1ω7c, and 18∶1ω5c, to represent the bacterial PLFAs [51], [52], [53], and the other three fatty acids (16∶1ω5c, 18∶2ω6.9c and 18∶1ω9c) as the fungal PLFAs [52], [53], [54]. We used the ratio of signature fungal and bacterial PLFAs as an indicator of soil microbial community structure [51], [53], [55]. The fatty acid profile of soil microbes was examined in soils collected in years 1, 3, 4, and 5.

### Statistical analysis

We examined results for the entire experimental duration from 2005–2009 (Year 1–Year 4), and used the linear mixed model [56] to test the main effects of CO_2_, O_3_ and the interaction of CO_2_ and O_3_, and whether these changed over time. We employed a set of covariance structures including compound symmetric model (CS), the first-order autoregressive model [AR (1)], and autoregressive with random effect to reduce autocorrelation. The *P* values for treatments and interaction terms were reported based on the covariance structure that minimized Akaike information criterion (AIC) and Bayesian information criterion (BIC) [56]. Data for soil and microbial parameters from mid-seasons (Appendix S1), plant and soil N contents, fungal and bacterial PLFAs and the fungi∶bacteria ratios were subjected to the analysis of variance using the mixed model. To test for relationships between variables, we conducted a correlation analysis using all the data generated over the 4-year period. A Chi-square (χ^2^) test was also conducted to examine whether the CO_2_ effect on microbial biomass, respiration and the community structure were correlated with the CO_2_ effect on N availability. We thus developed four contingency tables for SMR, MBC, MBN, and fungi∶bacteria ratio, respectively. All statistical analyses were performed using the SAS 9.1 (SAS Institute, Inc., Cary, NC, USA). For all tests, *P*≤0.05 was considered to indicate a statistically significant difference.

## Results

### Soybean N_2_ fixation and plant residue C and N inputs to soil

Elevated CO_2_ significantly increased symbiotic N_2_ fixation by soybean plants, while O_3_ decreased it. Over the 4-year period, total N derived from symbiotic N_2_ fixation in soybean was estimated at 92.5, 68.4, 119.1 and 109.7 g N m^−2^ in the CF, +O_3_, +CO_2_ and +CO_2_+O_3_ treatments, respectively. Compared to the CF treatment, the +CO_2_ treatment significantly increased the net N inputs to soil from symbiotic N_2_ fixation (excluding seed harvests) on average by 43%, while the +O_3_ treatment decreased it by 23% over the 4-year period (Table 2). However, there was no significant effect of CO_2_×O_3_ on the net N inputs derived from symbiotic N_2_ fixation in any year (Table 2).

**Table 2:** Effects of elevated CO_2_ and O_3_ on soybean N_2_ fixation and total N in the surface soil.

	Year 1	Year 2	Year 3	Year 4
N inputs to soil derived from soybean N_2_ fixation (g N m^−2^)				
Treatment				
CF	11.4±0.8	2.9±0.4	6.0±0.5	7.5±0.3
+O_3_	10.6±0.5	1.8±0.4	5.0±0.5	4.5±0.5
+CO_2_	15.1±0.4	4.8±0.2	8.3±0.7	10.0±0.5
+CO_2_+O_3_	14.7±1.4	3.8±0.2	7.8±0.3	8.6±0.7
Source				
O_3_	NS	**	NS	**
CO_2_	***	***	***	***
CO_2_×O_3_	NS	NS	NS	NS
Total soil N in the surface soil (0–5 cm) (g N m^−2^)				
Treatment				
CF	85.7±8.2	ND	ND	93.7±11.7
+O_3_	74.1±16.0	ND	ND	90.0±3.1
CO_2_	76.4±10.7	ND	ND	95.6±6.4
+CO_2_+O_3_	75.1±4.7	ND	ND	97.5±8.1

Values shown for N inputs to soil from N_2_ fixation exclude seeds.

Values are mean ± s.e.m. *** (*P*<0.001) and ** (*P*<0.01) denote statistically significant main treatment effects, ANOVA mixed models. ND, not determined. NS, not significant. CF, charcoal-filtered ambient air. +O_3_, elevated O_3_.+CO_2_, elevated CO_2_.+CO_2_+O_3_, elevated CO_2_+O_3_. The main treatment effects of CO_2_, O_3_ and the CO_2_×O_3_ interaction on soil N were not statistically significant for any years.

**Figure 1:**
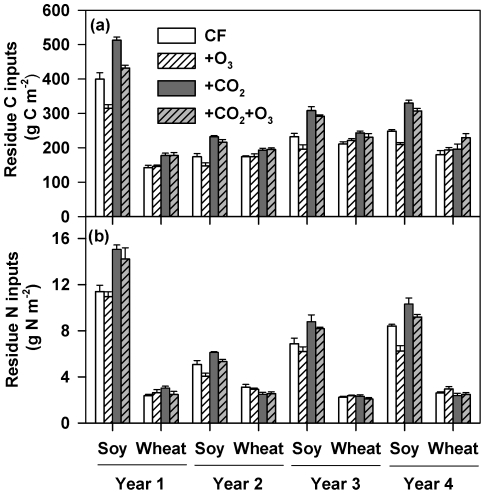
Aboveground residue C and N inputs under elevated CO_2_ and O_3_. Soy, soybean. CF, charcoal-filtered ambient air. +O_3_, elevated O_3_.+CO_2_, elevated CO_2_.+CO_2_+O_3_, elevated CO_2_+O_3_. Data represent means (n = 4) ± s.e.m. (a) Residue C inputs. Soybean residue C inputs: CO_2_ effect, *P*≤0.001 for every year; O_3_ effect, *P*<0.05 for every year; CO_2_×O_3_, *P*>0.1 for every year. Wheat residue C inputs: CO_2_ effect, *P*<0.05 for every year; O_3_ effect, *P*>0.1 for every year; CO_2_×O_3_, *P*>0.1 for every year, ANOVA mixed model. (b) Residue N inputs. Soybean residue N inputs: CO_2_ effect, *P*≤0.001 for every year; O_3_ effect, *P*<0.01 only for year 2 and 4; CO_2_×O_3_, *P*>0.1 for every year. Wheat residue N inputs: CO_2_ effect, *P*<0.05 for year 2 (significantly decreased) but >0.1 for year 1, 3 and 4; O_3_ effect, *P*>0.1 for every year; CO_2_×O_3_, *P*>0.1 for every year, ANOVA mixed model.

Elevated CO_2_ significantly increased both soybean aboveground residue C and N inputs to the chambers in all years (*P*≤0.001 for each year; Fig. 1), leading to an average increase by 38% and 30%, respectively, over the experimental period. Elevated CO_2_ also significantly increased wheat residue C inputs by 15%, but did not affect wheat residue N inputs (Fig. 1). Elevated O_3_ had no significant effects on wheat residue C and N inputs, but reduced soybean C and N inputs by 12% (Fig. 1). No significant CO_2_×O_3_ interaction was observed on soybean and wheat residue C and N inputs (*P*>0.1). Additionally, the total amounts of C and N in soybean residues were significantly different (*P*<0.01) among four years, which primarily resulted from the differences in biomass production of three different cultivars as well as the variability among years.

### Soil organic C and N, and extractable C and N

Over the experimental period, no significant CO_2_ or O_3_ effects on total soil organic C (data not shown) or total soil N (Table 2) were observed. However, there was a significant increase in total soil N over the experimental period in all treatments compared to the soil N before the treatments were applied in 2005. Although the magnitude of increase in soil N over time tended to be higher under elevated (26%) than ambient (17%) CO_2_ plots, this effect was not statistically significant.

Neither CO_2_ nor O_3_ treatments had any significant effects on soil extractable C in the whole soil profile or on the interactions between time and gas treatments over the 4-year period (Appendix S2). In general, elevated CO_2_ tended to increase concentrations of total extractable inorganic N (Appendix S3). Soil extractable N in elevated CO_2_ plots increased by, on average, 17% (*P*>0.1), 18% (*P*<0.01) and 8% (*P*>0.1), respectively, in 0–5, 5–10, and 10–20 cm soil layers over the 4-year period. There were no significant effects of O_3_ and the CO_2_×O_3_ interaction on soil extractable N (Appendix S2).

### Microbial biomass C and biomass N

**Figure 2:**
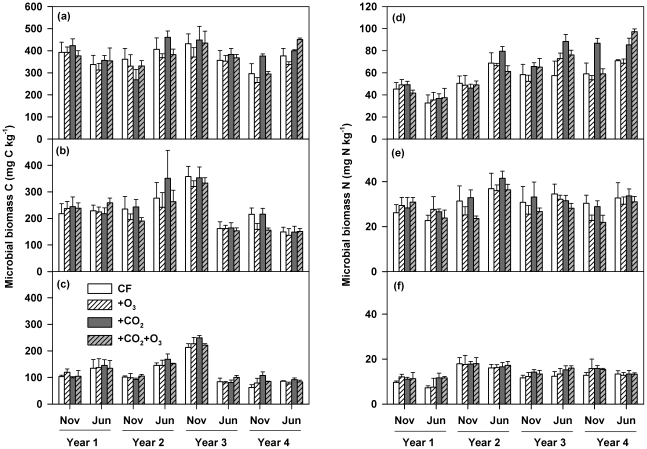
Effects of elevated CO_2_ and O_3_ on soil microbial biomass C and N. CF, charcoal-filtered ambient air. +O_3_, elevated O_3_.+CO_2_, elevated CO_2_.+CO_2_+O_3_, elevated CO_2_+O_3_. Microbial biomass C: (a) 0–5 cm soil layer (Repeated measures mixed model; CO_2_ effect: *P* = 0.026; CO_2_×Time: *P*>0.1), (b) 5–10 cm soil layer (Repeated measures mixed model; CO_2_ effect: *P*>0.1; CO_2_×Time: *P*>0.1) and (c) 10–20 cm soil layer (Repeated measures mixed model; CO_2_ effect: *P*>0.1; CO_2_×Time: *P*>0.1). Microbial biomass N: (d) 0–5 cm soil layer (Repeated measures mixed model; CO_2_ effect: *P* = 0.025; CO_2_×Time: *P* = 0.018), (e) 5–10 cm soil layer (Repeated measures mixed model; CO_2_ effect: *P*>0.1; CO_2_×Time: *P*>0.1) and (f) 10–20 cm soil layer (Repeated measures mixed model; CO_2_ effect: *P* = 0.040; CO_2_×Time: *P*>0.1). The O_3_ and CO_2_×O_3_ effects were not significant for all soil layers. Data represent means (n = 4) ± s.e.m.

Elevated CO_2_ significantly enhanced both MBC and MBN in the 0–5 cm soil layer, leading to an average increase of 8% (*P*<0.05; Fig. 2a) and 14% (*P*<0.05; Fig. 2d), respectively, over the 4-year period. However, these increases resulted primarily from CO_2_-induced enhancement in the third and fourth years of the experiment (Fig. 2). Both MBC and MBN at elevated CO_2_ remained unchanged in the top soil layer during year 1 and 2, but increased on average by 14% and 26%, respectively, within year 3 and 4 of the experiment. The CO_2_ effects were also significant for MBN in the 10–20 cm soil layer (*P*<0.05), but not significant for MBC (Appendix S2). However, neither O_3_ nor the CO_2_×O_3_ interaction had any significant impacts on MBC or MBN along the soil profile (Appendix S2).

### Soil microbial respiration (SMR)

**Figure 3:**
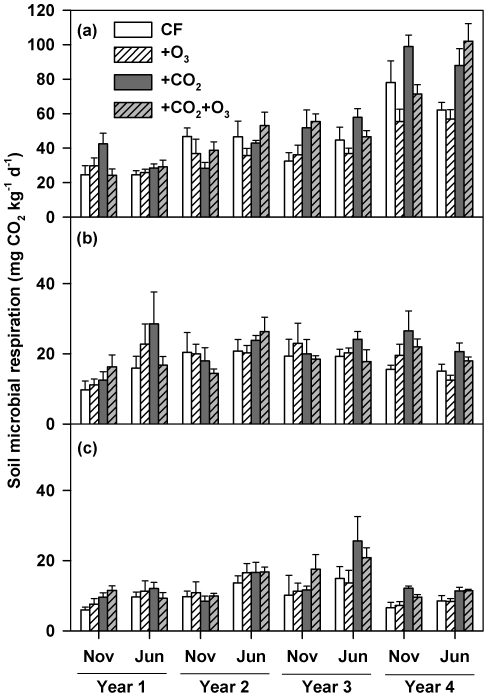
Effects of elevated CO_2_ and O_3_ on soil microbial respiration. CF, charcoal-filtered ambient air. +O_3_, elevated O_3_.+CO_2_, elevated CO_2_.+CO_2_+O_3_, elevated CO_2_+O_3_. (a) 0–5 cm soil layer (Repeated measures mixed model; CO_2_ effect: *P* = 0.012; CO_2_×Time: *P* = 0.003). (b) 5–10 cm soil layer (Repeated measures mixed model; CO_2_ effect: *P*>0.1; CO_2_×Time: *P*>0.1). (c) 10–20 cm soil layer (Repeated measures mixed model; CO_2_ effect: *P* = 0.044; CO_2_×Time: *P*>0.1). The O_3_ and CO_2_×O_3_ effects were not significant for all soil layers. Data represent means (n = 4) ± s.e.m.

Over the 4-year period, atmospheric CO_2_ enrichment increased SMR rates (Fig. 3). Compared to ambient CO_2_, SMR under elevated CO_2_ was 26% (*P*<0.05), 17% (*P*>0.1) and 31% (*P*<0.05) higher in 0–5, 5–10 and 10–20 cm soil layers, respectively. Similar to microbial biomass, the observed increases in SMR were largely due to the CO_2_ stimulation effects in the third and fourth years of the experiment (Fig. 3). In the 0–5 cm soil layer, for example, elevated CO_2_ only increased SMR by 9% in the first two years, but by 43% over the subsequent two years. Neither the O_3_ effect nor the CO_2_×O_3_ interaction resulted in significant effects on SMR in any soil layer (Appendix S2).

Metabolic quotient of soil microbes (the respiration rate per unit of microbial biomass C) under elevated CO_2_ increased on average by 16% (repeated measures mixed models; CO_2_ effect: *P* = 0.003), 9% (*P* = 0.2), and 20% (*P* = 0.02) in 0–5, 5–10, and 10–20 cm soil layers, respectively, over the 4-year period (Appendix S4). The CO_2_ effect on the metabolic quotient also changed considerably over time. In the 0–5 cm soil layer, CO_2_ enrichment slightly increased the metabolic quotient by 7% in the first two years, but significantly increased it by 25% within the following two years. Neither the O_3_ effect nor the CO_2_×O_3_ interaction resulted in significant impacts on metabolic quotient of soil microbes in any soil layer (Appendix S2).

### Net soil N mineralization

**Figure 4:**
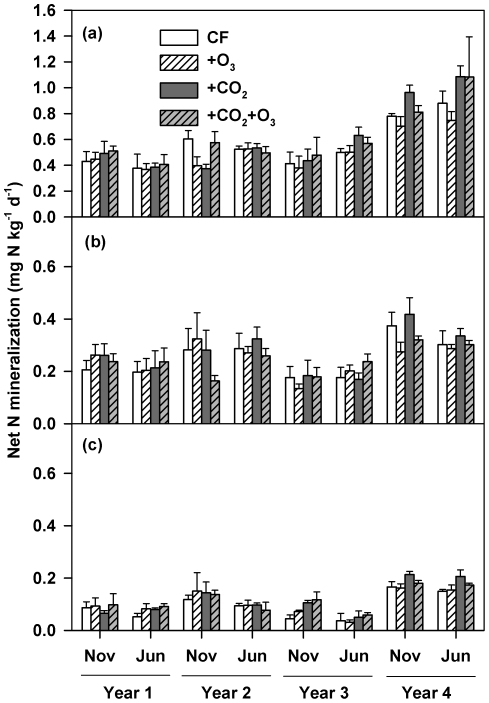
Effects of elevated CO_2_ and O_3_ on net soil N mineralization. CF, charcoal-filtered ambient air. +O_3_, elevated O_3_.+CO_2_, elevated CO_2_.+CO_2_+O_3_, elevated CO_2_+O_3_. (a) 0–5 cm soil layer (Repeated measures mixed model; CO_2_ effect: *P* = 0.002; CO_2_×Time: *P* = 0.011), (b) 5–10 cm soil layer (Repeated measures mixed model; CO_2_ effect: *P*>0.1; CO_2_×Time: *P*>0.1) and (c) 10–20 cm soil layer (Repeated measures mixed model; CO_2_ effect: *P* = 0.019; CO_2_×Time: *P*>0.1). The O_3_ and CO_2_×O_3_ effects were not significant for all soil layers. Data represent means (n = 4) ± s.e.m.

Similar to the effects on SMR and MBN, CO_2_ enrichment significantly stimulated the rate of net soil N mineralization at both 0–5 (*P*<0.01; Fig. 4a) and 10–20 (*P*<0.05; Fig. 4c) cm soil layers. On average, net mineralizable N (NMN) in elevated CO_2_ plots was 13%, 5%, and 26% higher than those in ambient CO_2_ plots, respectively, in the 0–5, 5–10, and 10–20 cm soil layers. Again, these effects were mainly due to the CO_2_-induced increases within the year 3 and 4 of the experiment (Fig. 4). Elevated CO_2_ showed no impacts on net soil N mineralization in the first two years, but caused an average increase by 22%, 12% and 49%, respectively, in the 0–5, 5–10, and 10–20 cm soil layers during the third and fourth years. In contrast, neither the O_3_ treatment effect nor the CO_2_×O_3_ interaction were statistically significant (Appendix S2).

### Stratification of soil microbial parameters under elevated CO_2_

The time course of CO_2_-effects on various parameters along the soil profile was significantly different. In the top 5-cm soil samples, MBC fluctuated over the whole period (Fig. 2a), but MBN, SMR and NMN started to increase by the third year (Fig. 2d, 3a and 4a). In the deeper soils (5–10 and 10–20 cm), MBC significantly decreased (Fig. 2b and 2c), MBN remained unchanged (Fig. 2e and 2f), but SMR and NMN increased (Fig. 3b, 3c, 4b and 4c) in years 3 and 4. Over the first two years of the experiment, all these parameters remained largely unaffected by CO_2_ enrichment in the 5–10 and 10–20 cm soil layers (Figs. 2, 3, 4). By the third and fourth years, elevated CO_2_ had no impacts on MBC in the deeper soil depths (Fig. 2b and 2c), but still increased SMR and NMN (Fig. 3b, 3c, 4b and 4c).

The microbial parameters from soil samples collected at the mid-seasons were similar with those at the harvest of the corresponding growing season and those results were shown in Appendix S1.

### PLFAs of soil microbes and the microbial community structure

**Figure 5:**
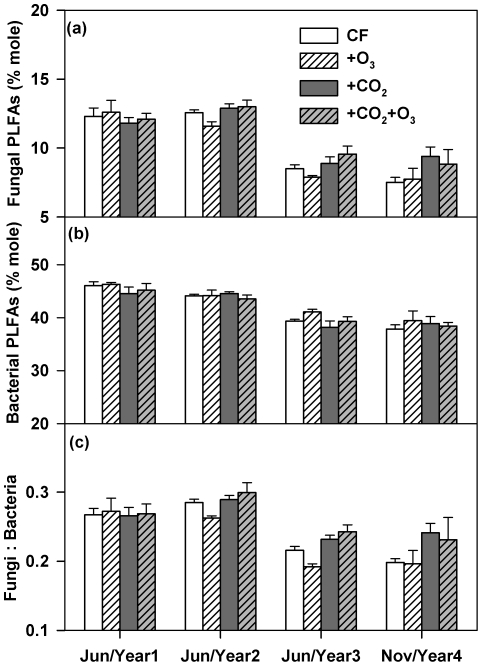
Effects of elevated CO_2_ and O_3_ on microbial community composition. CF, charcoal-filtered ambient air. +O_3_, elevated O_3_.+CO_2_, elevated CO_2_.+CO_2_+O_3_, elevated CO_2_+O_3_. (a) the abundance of fungal phospholipid fatty acids (PLFAs) [ANOVA mixed model; CO_2_ effect: Jun/Year 1 (*P*>0.1), Jun/Year 2 (*P* = 0.01), Jun/Year 3 (*P* = 0.029), Nov/Year 4 (*P* = 0.015); O_3_ effect: *P*>0.1 for all four time points; CO_2_×O_3_: *P*>0.1 for all four time points)], (b) the abundance of bacterial PLFAs (ANOVA mixed model; CO_2_ effect: *P*>0.1 for all four time points, O_3_ effect: *P*>0.1 for all four time points; CO_2_×O_3_: *P*>0.1 for all four time points) and (c) the ratio of fungal to bacterial PLFAs in top (0–5 cm) soils [ANOVA mixed model; CO_2_ effect: Jun/Year 1 (*P*>0.1), Jun/Year 2 (*P* = 0.006), Jun/Year 3 (*P*<0.001), Nov/Year 4 (*P* = 0.03); O_3_ effect: *P*>0.1 for all four time points; CO_2_×O_3_: Jun/Year 1 (*P*>0.1), Jun/Year 2 (*P* = 0.019), Jun/Year 3 (*P* = 0.032), Nov/Year 4 (*P*>0.1))]. Data represent means (n = 4) ± s.e.m.

Two trends in fungal and bacterial PLFAs emerged. First, the abundance of fungal and bacterial PLFAs and fungi∶bacteria ratios remained largely unchanged in the first two years but decreased significantly in years 4 and 5 of the experiment (Fig. 5). On average, fungal and bacterial PLFAs decreased by 31% and 13%, respectively, from the years 1–3 to years 4–5. Second, elevated CO_2_ significantly increased microbial PLFA biomass which was due only to increased fungal PLFA biomarkers starting from year 3 (*P*<0.05; Fig. 5a). As such, the fungi∶bacteria ratio increased significantly due to elevated CO_2_ (*P*<0.05; Fig. 5c). Neither the O_3_ effect nor the CO_2_×O_3_ interaction had significant effects on fungal and bacterial PLFAs at any time points (Fig. 5a, 5b).

### Correlation analysis

Correlation analysis, conducted among extractable C, extractable N, MBC, MBN, SMR and NMN, showed that all six parameters were significantly correlated with each other, though the coefficients varied considerably (Appendix S5). Net soil N mineralization can best explain the variation of MBC (*R* ^2^ = 0.44), MBN (*R* ^2^ = 0.72) and SMR (*R* ^2^ = 0.72). The χ^2^ values for MBC vs N, MBN vs N, SMR vs N and the fungi∶bacteria ratio vs N were 14.7 (*P*<0.001), 14.5 (*P*<0.001), 10.8 (*P*<0.01) and 4 (*P*<0.05) (Appendix S6), respectively, indicating that the CO_2_ effects on microbial biomass, activity and the community structure were closely related to CO_2_-induced alterations in N availability.

## Discussion

### The effect of elevated CO_2_ on soil microbes

Results obtained in this study showed that elevated CO_2_ influenced microbial processes over time likely through its impacts on C and N availability (Figs. 2, 3, 4, 5, Appendix S5 and F). Microbial responses to elevated CO_2_ have so far largely been considered in the context that soil microbes are C-limited [8], [11] while plant growth is N-limited [39], [57]. Many experiments have provided evidence that increased C availability induced by elevated CO_2_ enhanced soil microbial biomass and/or activities [5], [10], [11], [20], [26], [58] and can alter the structure of soil microbial communities in favor of fungal growth [16], [25], [55], [59]. In the current study, elevated CO_2_ significantly increased C and N availability for microbes by enhancing both aboveground (and presumably belowground) soybean and wheat residue C and N inputs in all four years (Fig. 1). However, microbial biomass, respiration and the community structure did not respond significantly until the third year (Fig. 2a, 2d, 3a, and 5c). Likely, it took a period of time for the increased residue inputs to accumulate to levels that affected soil microbial processes. It should be noted that the CO_2_-stimulation in soybean residue inputs was lower in year 2 compared with in other years (Fig. 1), which may have contributed to the time-lag in elevated CO_2_ effects on soil microbial processes observed in the present study.

Along with available C in soil, other factors such as soil moisture, soil food-web interactions and nutrient availability have also been suggested to affect microbial responses to elevated CO_2_, either singly or in combination [9], [11]. The availability of soil N has so far received the most attention in studies of elevated CO_2_ effects on soil microbes [9], [20], [21] because N is the most abundant nutrient element required for microbial growth [60]. The coincidence of higher microbial activities with increasing soil N availability and microbial biomass N in the CO_2_ treatments during the third and fourth years of the experiment suggests a link between soil N availability and microbial responses to elevated CO_2_ in this N-aggrading system. With the surface placement of residues in no-till systems, N existing in plant residues (mainly soybean) gradually moves into the soil profile, particularly the top soil layer, through leaching and decomposition processes. Higher N inputs from residues of plants grown under elevated CO_2_ (Fig. 1b), which stemmed from both CO_2_-stimulation of N_2_ fixation (Table 2) [35], [36] and possibly plant N retention [4], [61], can in turn increase soil N availability for microbes. In a recent meta-analysis of 131 manipulation studies with tree species, Dieleman *et al*. (2010) found that the CO_2_-enhancement of microbial activity and decomposition was positively correlated with increasing soil N availability. These results are similar to the CO_2_-stimulation of microbial growth and activities along with increasing available soil N observed in our study. Also, it has been well documented that mineral N additions can stimulate decomposition of plant residues, particularly the non-lignin components [28], [62]; thus increased soil N availability can significantly facilitate decomposition of non-lignin components of crop residues. All exoenzymes responsible for disintegrating organic materials are N-rich proteins, and sufficient supplies of N for microbes may facilitate enzyme production [28], [63], though addition of inorganic N could also suppress lignin-degrading enzymes [64]. Other studies have also recently showed that N addition stimulated microbial respiration [26] and decomposition activities [25].

Changes in fungal and bacterial PLFAs, and their ratios observed in our study provide new insights into how alterations in the relative availability of C and N can modulate microbial activities and their responses to elevated CO_2_. First, the coincidence between decreased fungi∶bacteria ratios in years 4 and 5 in comparison with previous years and increased N availability and MBN over time is consistent with the general concept that high N availability favors bacteria over fungi [28], [29]. Evidently, high N inputs due to N fertilization of wheat and soybean N_2_ fixation in our system gradually increased soil N availability and altered the soil microbial community composition over time. In a loblolly pine system, Feng *et al*. (2010) also observed that N fertilization reduced the fungi∶bacteria ratio. Second, significantly higher fungi∶bacteria ratios under elevated than ambient CO_2_ indicate that CO_2_-enhancement of C inputs may still play a major role in shaping the community structure in N-rich agroecosysystems, as shown in many forests and grasslands [4], [16], [25], [55], [59], [65]. What was surprising is that the significant increases in microbial respiration (Fig. 3a) concurred with decreased microbial PLFA biomass (Fig. 5a, 5b), leading to an increase in metabolic quotient over time as well as under elevated CO_2_ (Appendix S4). Since both fungi and bacteria identified by PLFAs represent the most active part of soil microorganisms [52], [59], these results indicate that high N inputs may have stimulated microbial physiological activities and/or microbial biomass turnover. Taken together, our results suggest that CO_2_-induced changes in soil N availability might be an important factor that concurrently mediated elevated CO_2_ effects on soil microbes and microbial feedbacks in this N-aggrading agroecosystem.

The findings that the stimulation of soil microbes under elevated CO_2_ over the course of the experiment may have significant implications for understanding residue turnover and soil C sequestration in agroecosystems under future climate change scenarios. In many natural and semi-natural ecosystems, the CO_2_-induced stimulation of plant growth may not persist because of nutrient limitation [9], [39]. In agricultural ecosystems, however, N is typically not a limiting factor for plant growth due to the application of chemical N fertilizers and/or the incorporation of legume plants, and CO_2_-stimulation of biomass production is expected to be sustained [2], [31], [66]. Therefore, it has been suggested that elevated CO_2_ can increase long-term C storage in agroecosystems, particularly in combination with no-tillage management [38], [67], [68]. However, this assumption does not fully consider the C output from agroecosystems: unlike forest ecosystems where the standing biomass constitutes a major C pool, most agroecosystems must accumulate C in the soil for ecosystem C sequestration to occur. Consequently, the fate of returning residues will largely determine the potential of agroecosystem C sequestration. The close correlations between N availability and both microbial respiration and metabolic quotient under elevated CO_2_ in our study (Fig. 3, Appendix S4 and Appendix S6) indicate that soil microbes became more active with CO_2_ enrichment. Our results, along with other previous findings [27], suggest that high N availability may significantly increase soil organic C turnover in agroecosystems through stimulating residue decomposition under future CO_2_ scenarios, highlighting the need to examine the interactive effect of soil N availability and atmospheric CO_2_ on soil organic C dynamics.

It is also interesting to note that microbial parameters along the soil profile exhibited different patterns under elevated CO_2_ (Figs. 2, 3, 4). No-till systems are characterized by vertical stratification of soil organic C and microbial biomass because of continuous residue surface placement [37], [69]. Rapid deceases in MBC and diminished CO_2_ effects on MBC starting in the third year in deeper soil layers (Fig. 2b and 2c) seems to suggest that alteration in C availability caused by residue placement may dominate microbial responses. However, other parameters [MBN (Fig. 2f), SMR (Fig. 3c) and NMN (Fig. 4c)] did not decrease correspondingly with MBC and continued to significantly respond to elevated CO_2_ (Appendix S2), suggesting that other factors may significantly exert control. High correlations between MBN and SMR, and NMN in deeper soil depths (Appendix S5) suggest that N availability critically modulated microbial activities. In no-till systems, root-derived C is the primary source for deep soil C and CO_2_-stimulation of both fine and deep roots has been proposed as a potential mechanism that facilitates C sequestration there [37]. However, higher metabolic quotient (Appendix S4), SMR (Fig. 3) and NMN (Fig. 4) under elevated CO_2_ indicate that not only were microbes more active but also organic C turnover was more rapid in the deeper soil layers. Consequently, high root production under elevated CO_2_ might stimulate C losses from deep soil layers by priming decomposition of indigenous organic matter [15], [58], [66], [70], [71]. Long-term experiments are critically needed to examine whether the stimulation of SMR and NMN in our study is transient or will be sustained over time.

### Effects of elevated O_3_ and CO_2_×O_3_ on soil microbes

Elevated O_3_ often leads to a substantial decline in the aboveground biomass of O_3_-sensistive plants [7], [30], [72] and subsequent C allocation belowground [6], [19]. In the current study, the statistically significant decline in plant residue C primarily stemmed from O_3_-reduction of soybean residue C (by 12% on average; *P*<0.05). The unresponsiveness of wheat to O_3_ was likely due to the relatively low O_3_ concentrations during the wheat growing season (Table 1), use of a relatively O_3_-tolerant cultivar, and possibly other environmental conditions (for example, temperature and light levels). Ambient O_3_ concentrations during winter wheat growing seasons are usually low due to the lower concentrations of precursors of O_3_ formation and the lower temperatures during the winter and the early spring. The decrease in soybean residue N inputs under elevated O_3_ (Fig. 1b) resulted from O_3_-induced reduction in residue biomass and possibly symbiotic N_2_ fixation in soybean plants (Table 2) [36]. However, no significant O_3_ effects were detected on any soil microbial parameters in this study (Figs. 2, 3, 4, 5, Appendix S2). These results suggest that N inputs through both fertilization and N_2_ fixation in our system might overtake O_3_-induced reduction of residue N in affecting soil microbes. Alternatively, these results also suggest that the magnitude of reductions in both C and N under elevated O_3_ were insufficient to substantially affect soil microbial activity in our experiment. In an OTC experiment under conventional tillage practice, Islam *et al.* (2000) also found that elevated O_3_ had no significant impacts on soil microbial respiration.

Our results showed that elevated O_3_ tended to reduce soybean residue C and N inputs under elevated CO_2_ (Fig. 1). This indicated that added O_3_ prevented a portion of the CO_2_-induced stimulation in biomass production from occurring. Such a pattern, however, was not observed for microbial parameters over the course of the experiment (Figs. 2, 3, 4, 5). The lack of microbial responses to O_3_ under elevated CO_2_ suggests that, as noted above, the magnitude of the combination of elevated CO_2_ and O_3_ effect on residue C and N inputs was not enough to influence soil microbes in the current study. It is also possible that O_3_ might not necessarily diminish the stimulation effect of elevated CO_2_ on C allocation belowground through fine root biomass, root exudation and turnover during plant growth, as observed in the Rhinelander free-air CO_2_ and O_3_ enrichment study using tree species [73]. Regardless of the underlying causes, our results suggest that O_3_ may have limited impact on soil microbial processes in agricultural systems under future CO_2_ scenarios and that its effect will be dependent on the sensitivity of crop cultivars to O_3_.

### Conclusions

In summary, results obtained from this study showed that the responses of soil microbes and their community structure to elevated CO_2_ significantly changed through time in the N-aggrading wheat-soybean rotation system, and that these may be largely related to CO_2_-induced alterations in soil C and N availability. While soil microbial biomass, activities and the community structure composition were little affected by elevated CO_2_ in the first two years, they significantly responded to CO_2_ enrichment in the third and fourth years of the experiment as N availability increased. However, O_3_ effects on soil C and N availability were likely insufficient in magnitude to produce detectable changes in the soil microbial parameters measured. Together, these results highlight the urgent need for considering the interactive impact of C and N availability on microbial activities and decomposition when projecting soil C balance in N-rich systems under future CO_2_ scenarios.

## Supporting Information

Appendix S1 Effects of CO_2_ enrichment on soil microbial parameters during mid-growing seasons.
(DOCX)

Appendix S2 *P* values of analyses of repeated measures linear mixed models of CO_2_, O_3_ and time effects, and all interactions over 4 years.
(DOCX)

Appendix S3 Effects of elevated CO_2_ and O_3_ on soil extractable N.
(TIF)

Appendix S4 Effects of elevated CO_2_ and O_3_ on metabolic quotient of soil microbes.
(TIF)

Appendix S5 Linear correlations among microbial respiration, microbial biomass C and N, extractable C and N, net N mineralization of soils over the 4-year period.
(DOCX)

Appendix S6 Chi-square test of relationship between the CO_2_ effect on N availability and the CO_2_ effect on microbial biomass, respiration and the community structure.
(DOCX)
