# Supplementary material for: Soil Microbial Responses to Elevated CO2 and O3 in a Nitrogen-Aggrading Agroecosystem
Source: PLoS One. 2011 Jun 22;6(6):e21377. doi: 10.1371/journal.pone.0021377 (PMC3120872; doi:10.1371/journal.pone.0021377)
Supplement: Appendix S1 — Effects of CO2 enrichment on soil microbial parameters during mid-growing seasons. (DOCX) [file pone.0021377.s001.docx]

**Appendix S1** Effects of elevated CO_2_ on soil microbial respiration, microbial biomass C and N, extractable C and inorganic N, and net N mineralization during mid-growing seasons.

| Month | Year | 0-5 cm | |  | 5-10 cm | |
| --- | --- | --- | --- | --- | --- | --- |
|  |  | Ambient CO_2_ | Elevated CO_2_ |  | Ambient CO_2_ | Elevated CO_2_ |
| Soil microbial respiration (mg CO_2_ kg^-1^ d^-1^) | | | | | | |
| April | 1 | 28.9 (2.8) | 37.7 (4.1) |  | 19.0 (2.4) | 20.3 (1.7) |
| April | 2 | 51.6 (4.4) | 59.1 (4.4) |  | 28.4 (1.3) | 30.0 (2.1) |
| August | 3 | 51.5 (1.7) | 52.5 (2.9) |  | ND | ND |
| August | 4 | 51.5 (4.8) | 91.1 (9.5)** |  | 24.7 (1.6) | 37.7 (3.0)* |
| Microbial biomass C (mg C kg^-1^) | | | | | | |
| April | 1 | 394 (25) | 457 (26) |  | 264 (24) | 270 (17) |
| April | 2 | 488 (54) | 521 (56) |  | 162 (9) | 161 (8) |
| August | 3 | 430 (36) | 484 (24) |  | ND | ND |
| August | 4 | 239 (13) | 321 (18)** |  | 139 (10) | 142 (9) |
| Microbial biomass N (mg N kg^-1^) | | | | | | |
| April | 1 | 43.3 (2.5) | 52.7 (3.7) |  | 24.8 (2.7) | 25.8 (2.8) |
| April | 2 | 53.1 (3.6) | 57.8 (3.5) |  | 28.4 (2.3) | 28.8 (1.4) |
| August | 3 | 52.7 (3.4) | 54.3 (3.1) |  | ND | ND |
| August | 4 | 51.6 (2.3) | 69.5 (4.6)** |  | 28.4 (1.6) | 28.4 (1.7) |
| Extractable organic C (mg C kg^-1^) | | | | | | |
| April | 1 | 77.8 (3.3) | 79.6 (5.7) |  | 70.8 (4.0) | 69.0 (5.4) |
| April | 2 | 67.4 (3.5) | 73.5 (3.8) |  | 58.2 (4.6) | 55.1 (4.3) |
| August | 3 | 84.5 (5.2) | 84.3 (5.1) |  | ND | ND |
| August | 4 | 76.0 (4.3) | 78.2 (2.6) |  | 50.2 (2.8) | 51.4 (2.9) |
| Extractable inorganic N (mg N kg^-1^) | | | | | | |
| April | 1 | 17.7 (2.4) | 13.4 (1.8) |  | 12.7 (1.8) | 11.9 (2.0) |
| April | 2 | 7.3 (0.5) | 8.0 (0.5) |  | 4.2 (0.3) | 4.0 (0.3) |
| August | 3 | 6.0 (0.3) | 7.2 (0.3)* |  | ND | ND |
| August | 4 | 7.7 (0.2) | 9.5 (0.4)** |  | 3.9 (0.2) | 4.6 (0.3) |
| Net N mineralization (mg N kg^-1^ d^-1^) | | | | | | |
| April | 1 | 0.52 (0.04) | 0.63 (0.04) |  | 0.18 (0.02) | 0.22 (0.02) |
| April | 2 | 0.55 (0.04) | 0.55 (0.03) |  | 0.28 (0.02) | 028 (0.02) |
| August | 3 | 0.41 (0.02) | 0.43 (0.02) |  | ND | ND |
| August | 4 | 0.67(0.05) | 0.87 (0.03)* |  | 0.33 (0.02) | 0.36 (0.03) |

ND: not determined. April/ Year 1: wheat season, April/Year 2: wheat season, August/ Year 3: soybean season, August/Year 4: soybean season. *(*P* < 0.05) or ** (*P* < 0.01) denote statistically significant effects of CO_2_ treatment at a specific sampling date, ANOVA.
