# Supplementary material for: Soil Microbial Responses to Elevated CO2 and O3 in a Nitrogen-Aggrading Agroecosystem
Source: PLoS One. 2011 Jun 22;6(6):e21377. doi: 10.1371/journal.pone.0021377 (PMC3120872; doi:10.1371/journal.pone.0021377)
Supplement: Appendix S2 — P values of analyses of repeated measures linear mixed models of CO2, O3 and time effects, and all interactions over 4 years. (DOCX) [file pone.0021377.s002.docx]

**Appendix S2** *P* values of analyses of repeated measures linear mixed models of CO_2_, O_3_ and time effects, and all interactions over 4 years. Significant effects (*P* ≤ 0.05) are shown in bold text.

| Variable | Depth (cm) | CO_2_ | O_3_ | Time | CO_2_ × O_3_ | Time × CO_2_ | Time × O_3_ | Time ×  CO_2_ × O_3_ |
| --- | --- | --- | --- | --- | --- | --- | --- | --- |
|  |  |  |  |  |  |  |  |  |
| Microbial respiration | 0-5 | **0.012** | 0.160 | **< 0.001** | 0.469 | **0.003** | **0.046** | 0.179 |
|  | 5-10 | 0.149 | 0.615 | **0.017** | 0.159 | 0.412 | 0.922 | 0.449 |
|  | 10-20 | **0.044** | 0.576 | **< 0.001** | 0.817 | 0.121 | 0.853 | 0.965 |
|  |  |  |  |  |  |  |  |  |
| Microbial biomass C | 0-5 | **0.026** | 0.140 | **< 0.001** | 0.558 | 0.319 | 0.838 | 0.739 |
|  | 5-10 | 0.429 | 0.138 | **< 0.001** | 0.883 | 0.961 | 0.505 | 0.975 |
|  | 10-20 | 0.226 | 0.856 | **< 0.001** | 0.397 | 0.803 | 0.958 | 0.740 |
|  |  |  |  |  |  |  |  |  |
| Microbial biomass N | 0-5 | **0.025** | 0.630 | **< 0.001** | 0.135 | **0.018** | 0.174 | 0.643 |
|  | 5-10 | 0.820 | 0.080 | **0.002** | 0.445 | 0.940 | 0.349 | 0.990 |
|  | 10-20 | **0.040** | 0.526 | **< 0.001** | 0.578 | 0.810 | 0.998 | 0.995 |
|  |  |  |  |  |  |  |  |  |
| Extractable organic C | 0-5 | 0.094 | 0.457 | **< 0.001** | 0.366 | 0.781 | 0.759 | 0.953 |
|  | 5-10 | 0.907 | 0.254 | **< 0.001** | 0.106 | 0.988 | 0.990 | 0.968 |
|  | 10-20 | 0.528 | 0.266 | **< 0.001** | 0.054 | 0.985 | 0.971 | 0.998 |
|  |  |  |  |  |  |  |  |  |
| Extractable inorganic N | 0-5 | 0.107 | 0.270 | **< 0.001** | 0.328 | 0.749 | 0.933 | 0.791 |
|  | 5-10 | **0.004** | 0.386 | **< 0.001** | 0.248 | 0.176 | 1.000 | 0.955 |
|  | 10-20 | 0.430 | 0.905 | **< 0.001** | 0.871 | 0.088 | 0.908 | 0.891 |
|  |  |  |  |  |  |  |  |  |
| Net N mineralization | 0-5 | **0.002** | 0.940 | **< 0.001** | 0.796 | **0.011** | 0.949 | 0.518 |
|  | 5-10 | 0.426 | 0.422 | **< 0.001** | 0.322 | 0.313 | 0.692 | 0.700 |
|  | 10-20 | **0.019** | 0.681 | **< 0.001** | 0.350 | 0.453 | 0.926 | 0.974 |
