# Supplementary figures and images for: Soil Microbial Responses to Elevated CO2 and O3 in a Nitrogen-Aggrading Agroecosystem
Source: PLoS One. 2011 Jun 22;6(6):e21377. doi: 10.1371/journal.pone.0021377 (PMC3120872; doi:10.1371/journal.pone.0021377)

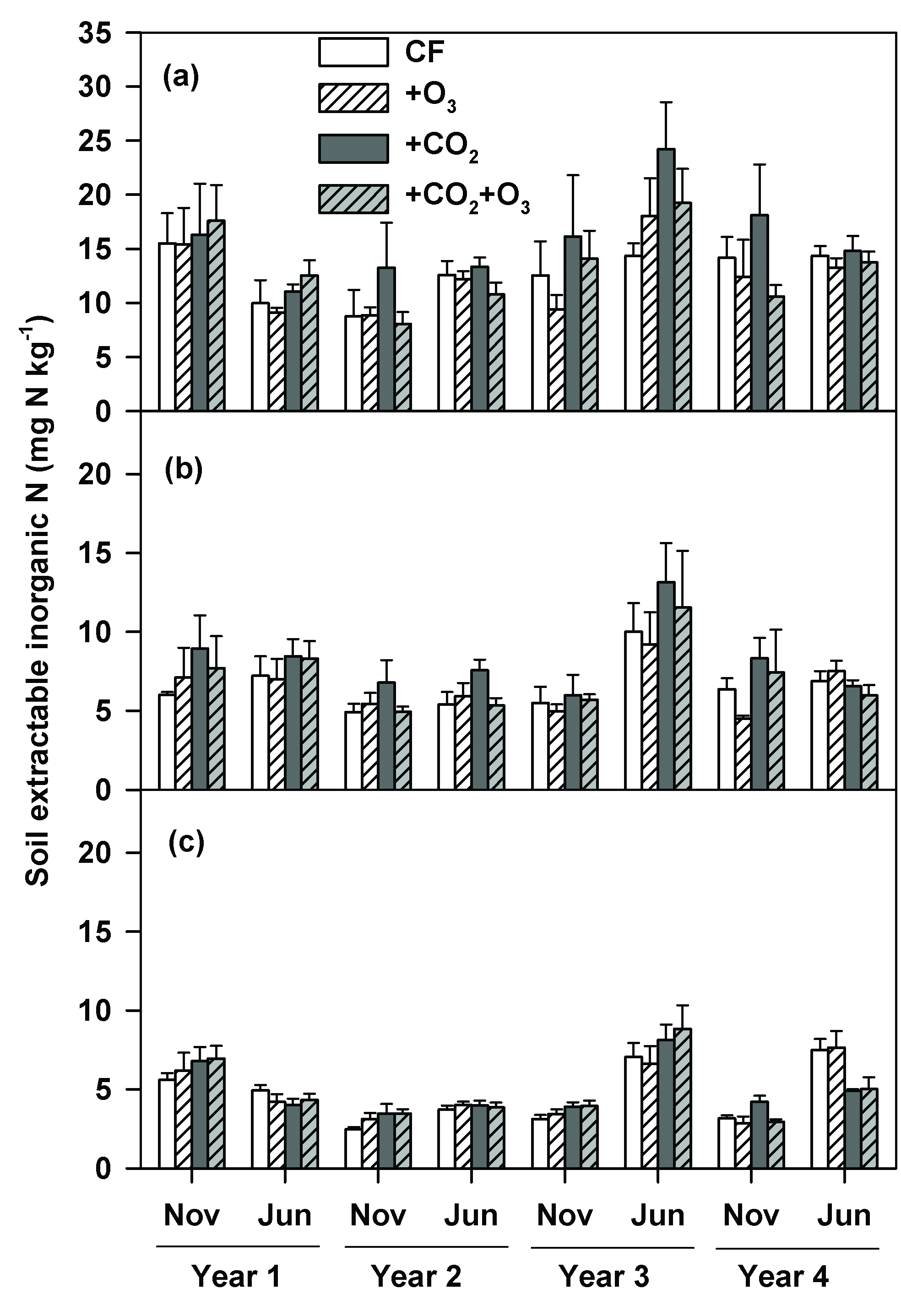

Supplement: Appendix S3 — Effects of elevated CO2 and O3 on soil extractable N. (TIF) [file pone.0021377.s003.tif]

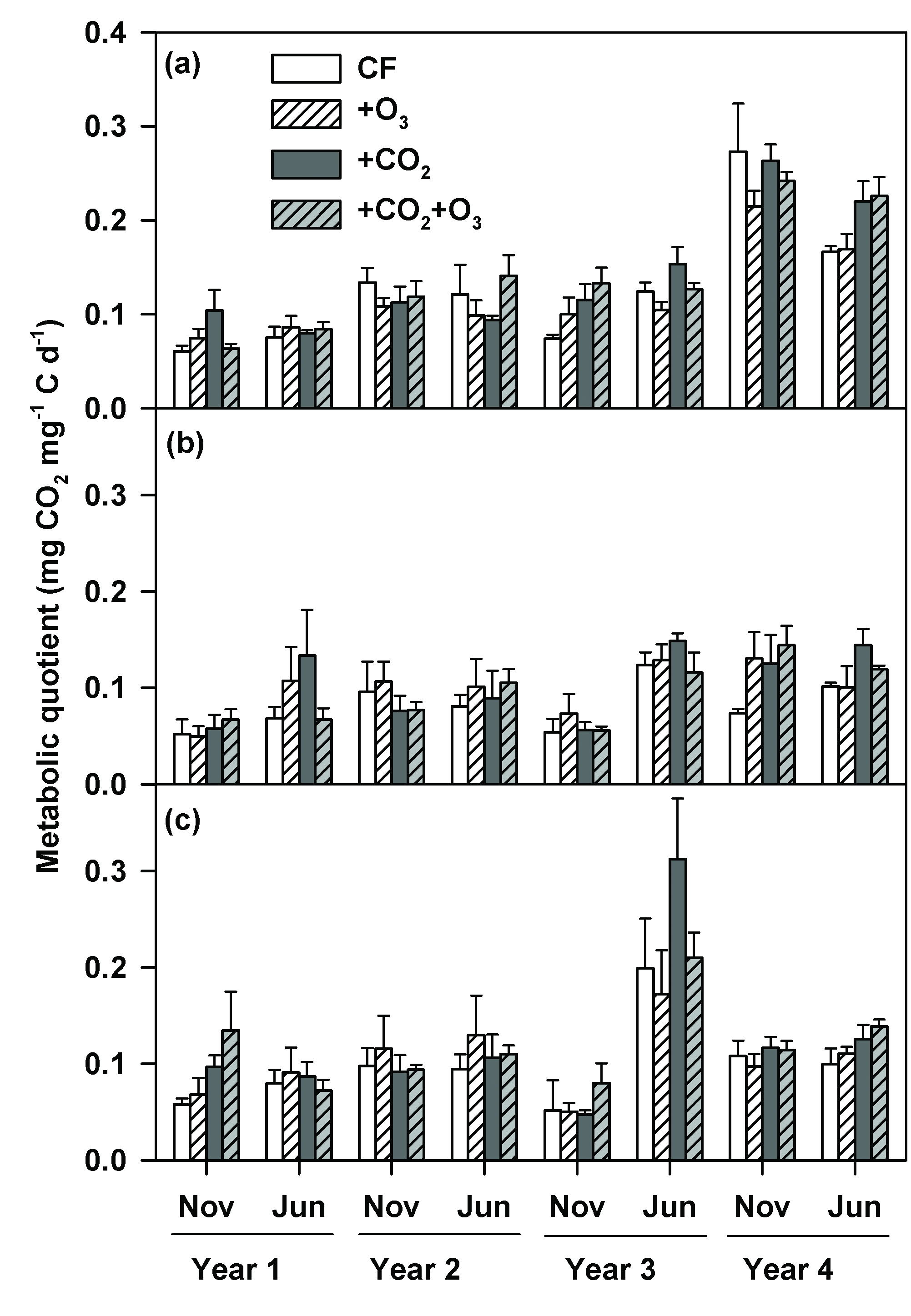

Supplement: Appendix S4 — Effects of elevated CO2 and O3 on metabolic quotient of soil microbes. (TIF) [file pone.0021377.s004.tif]
