# Supplementary material for: Soil Microbial Responses to Elevated CO2 and O3 in a Nitrogen-Aggrading Agroecosystem
Source: PLoS One. 2011 Jun 22;6(6):e21377. doi: 10.1371/journal.pone.0021377 (PMC3120872; doi:10.1371/journal.pone.0021377)
Supplement: Appendix S5 — Linear correlations among microbial respiration, microbial biomass C and N, extractable C and N, net N mineralization of soils over the 4-year period. (DOCX) [file pone.0021377.s005.docx]

**Appendix S5** Linear correlations (*R*) among microbial respiration, microbial biomass C and N, extractable C and N, net N mineralization of soils over the 4-year period.

|  | SMR | MBC | MBN | Extr-C | Extr-N | NMN |
| --- | --- | --- | --- | --- | --- | --- |
| SMR | 1 | 0.636*** | 0.826*** | 0.419*** | 0.605*** | 0.847*** |
| MBC |  | 1 | 0.809*** | 0.511*** | 0.581*** | 0.664*** |
| MBN |  |  | 1 | 0.512*** | 0.687*** | 0.846*** |
| Extr-C |  |  |  | 1 | 0.565*** | 0.449*** |
| Extr-N |  |  |  |  | 1 | 0.581*** |
| NMN |  |  |  |  |  | 1 |

*** denotes significance at *P* < 0.0001 (n = 384). SMR, soil microbial respiration; MBC, microbial biomass C; MBN, microbial biomass N; Extr-C, soil extractable organic C; Extr-N, total soil extractable inorganic N; NMN, net N mineralization.
