# Supplementary material for: Soil Microbial Responses to Elevated CO2 and O3 in a Nitrogen-Aggrading Agroecosystem
Source: PLoS One. 2011 Jun 22;6(6):e21377. doi: 10.1371/journal.pone.0021377 (PMC3120872; doi:10.1371/journal.pone.0021377)
Supplement: Appendix S6 — Chi-square test of relationship between the CO2 effect on N availability and the CO2 effect on microbial biomass, respiration and the community structure. (DOCX) [file pone.0021377.s006.docx]

**Appendix S6** Chi-square test (χ^2^) of relationship between the CO_2_ effect on N availability and the CO_2_ effect on microbial biomass, respiration and the community structure. For microbial biomass C, biomass N and microbial respiration, we collected 34 observations including three from the fifth year (data not shown). For the fungi:bacteria ratio, we only collected 4 observations. Values in the four cells of the contingency table are the frequency of observations. *P* < 0.05 indicates a significant relationship between the CO_2_-induced changes in N availability and the elevated CO_2_ effect on microbial parameters.

Table S6a Contingency table for microbial biomass C vs N availability.

| **N availability** | **Microbial biomass C** | | |
| --- | --- | --- | --- |
|  | Difference | No difference | Total |
| **Difference** | 4 | 0 | 4 |
| **No difference** | 4 | 26 | 30 |
| **Total** | 8 | 26 | 34 |

χ^2^ =14.73, *P* < 0.001

Table S6b Contingency table for microbial biomass N vs N availability.

| **N availability** | **Microbial biomass N** | | |
| --- | --- | --- | --- |
|  | Difference | No difference | Total |
| **Difference** | 5 | 3 | 8 |
| **No difference** | 1 | 25 | 26 |
| **Total** | 6 | 28 | 34 |

χ^2^ =14.48, *P* < 0.001

Table S6c Contingency table for microbial respiration vs N availability.

| **N availability** | **Microbial respiration** | | |
| --- | --- | --- | --- |
|  | Difference | No difference | Total |
| **Difference** | 7 | 1 | 8 |
| **No difference** | 6 | 20 | 26 |
| **Total** | 13 | 21 | 34 |

χ^2^ =10.75, *P* = 0.001

**Table S6** **continued**

Table S6d Contingency table for the fungi:bacteria ratio vs N availability.

| **N availability** | **Fungi:bacteria ratio** | | |
| --- | --- | --- | --- |
|  | Difference | No difference | Total |
| **Difference** | 3 | 0 | 3 |
| **No difference** | 0 | 1 | 1 |
| **Total** | 3 | 1 | 4 |

χ^2^ =4, *P* = 0.046
